# Supplementary material for: The East Asian gut microbiome is distinct from colocalized White subjects and connected to metabolic health
Source: eLife. 2021 Oct 7;10:e70349. doi: 10.7554/eLife.70349 (PMC8612731; doi:10.7554/eLife.70349)
Supplement: Transparent reporting form [file elife-70349-transrepform1.docx]

***eLife’s* transparent reporting form**

We encourage authors to provide detailed information *within their submission* to facilitate the interpretation and replication of experiments. Authors can upload supporting documentation to indicate the use of appropriate reporting guidelines for health-related research (see [EQUATOR Network](http://www.equator-network.org/%20)), life science research (see the [BioSharing Information Resource](https://biosharing.org/)), or the [ARRIVE guidelines](http://www.plosbiology.org/article/info:doi/10.1371/journal.pbio.1000412) for reporting work involving animal research. Where applicable, authors should refer to any relevant reporting standards documents in this form.

If you have any questions, please consult our Journal Policies and/or contact us: [editorial@elifesciences.org](mailto:editorial@elifesciences.org).

**Sample-size estimation**

- You should state whether an appropriate sample size was computed when the study was being designed
- You should state the statistical method of sample size computation and any required assumptions
- If no explicit power analysis was used, you should describe how you decided what sample (replicate) size (number) to use

Please outline where this information can be found within the submission (e.g., sections or figure legends), or explain why this information doesn’t apply to your submission:

No explicit power calculation was done *a priori* for the human or mouse study designs. All participants who were successfully enrolled and recruited to the IDEO cohort at the time of sampling processing were included for microbiome analyses. In gnotobiotic experiments, matched humans were selected as described in the Methods Section and Supplementary file 1H. As prompted by this form, we conducted G*Power 3.1 power calculation of an effect size of ~10 for Bacteroides (a unifying and consistent signal seen in multiple experiments) results in an estimated total sample size of 4 for a Wilcoxon-Mann-Whitney p < 0.05 and Beta error of 0.95. For microbial beta-diversity, we employed PERMANOVA testing utilizing adonis in vegan, which is a permutational test and ideal for handling microbial datasets.

**Replicates**

- You should report how often each experiment was performed
- You should include a definition of biological versus technical replication
- The data obtained should be provided and sufficient information should be provided to indicate the number of independent biological and/or technical replicates
- If you encountered any outliers, you should describe how these were handled
- Criteria for exclusion/inclusion of data should be clearly stated
- High-throughput sequence data should be uploaded before submission, with a private link for reviewers provided (these are available from both GEO and ArrayExpress)

Please outline where this information can be found within the submission (e.g., sections or figure legends), or explain why this information doesn’t apply to your submission:

The human cohort represents one cross-sectional human study wherein biological replicates represent separate individuals. The gnotobiotic mouse experimental design is shown in Figure 7-figure supplement 1; we separately specify the number of donor and recipient mice. All sequencing datasets represent separate biological replicates. As described below, sequencing data is available in the NIH Sequence Read Archive using accession PRJNA665061.

**Statistical reporting**

- Statistical analysis methods should be described and justified
- Raw data should be presented in figures whenever informative to do so (typically when N per group is less than 10)
- For each experiment, you should identify the statistical tests used, exact values of N, definitions of center, methods of multiple test correction, and dispersion and precision measures (e.g., mean, median, SD, SEM, confidence intervals; and, for the major substantive results, a measure of effect size (e.g., Pearson's r, Cohen's d)
- Report exact p-values wherever possible alongside the summary statistics and 95% confidence intervals. These should be reported for all key questions and not only when the p-value is less than 0.05.

Please outline where this information can be found within the submission (e.g., sections or figure legends), or explain why this information doesn’t apply to your submission:

Statistical methods are described in the Methods Section labelled “Statistical analyses”. Statistical tests used, exact values of N and other relevant details are also included in the figure legends. Exact p-values are reported wherever possible.

(For large datasets, or papers with a very large number of statistical tests, you may upload a single table file with tests, Ns, etc., with reference to sections in the manuscript.)

**Group allocation**

- Indicate how samples were allocated into experimental groups (in the case of clinical studies, please specify allocation to treatment method); if randomization was used, please also state if restricted randomization was applied
- Indicate if masking was used during group allocation, data collection and/or data analysis

Please outline where this information can be found within the submission (e.g., sections or figure legends), or explain why this information doesn’t apply to your submission:

This is a cross sectional human cohort, and inclusion and exclusion criteria are described in the Methods section. Masking was not utilized during data collection or data analysis.

**Additional data files (“source data”)**

- We encourage you to upload relevant additional data files, such as numerical data that are represented as a graph in a figure, or as a summary table
- Where provided, these should be in the most useful format, and they can be uploaded as “Source data” files linked to a main figure or table
- Include model definition files including the full list of parameters used
- Include code used for data analysis (e.g., R, MatLab)
- Avoid stating that data files are “available upon request”

Please indicate the figures or tables for which source data files have been provided:

Data relevant for complete reproduction of this analysis is provided in Supplementary file 1. As described in our data availability section, sequencing data is available at the NIH Sequence Read Archive under accession PRJNA665061. All code for this manuscript is available on request and if not requested during review will be deposited at the following site prior to publication: https://github.com/turnbaughlab/2021_IDEO.
